# Quality and Safety Considerations for Therapeutic Products Based on Extracellular Vesicles

**DOI:** 10.1007/s11095-024-03757-4

**Published:** 2024-08-07

**Authors:** Yoshinobu Takakura, Rikinari Hanayama, Kazunari Akiyoshi, Shiroh Futaki, Kyoko Hida, Takanori Ichiki, Akiko Ishii-Watabe, Masahiko Kuroda, Kazushige Maki, Yasuo Miura, Yoshiaki Okada, Naohiro Seo, Toshihide Takeuchi, Teruhide Yamaguchi, Yusuke Yoshioka

**Affiliations:** 1 Department of Biopharmaceutics and Drug Metabolism, Graduate School of Pharmaceutical Sciences, Kyoto University, Kyoto, Japan; 2 WPI Nano Life Science Institute (NanoLSI), Kanazawa University, Kanazawa, Japan; 3 Department of Polymer Chemistry, Graduate School of Engineering, Kyoto University, Kyoto, Japan; 4 Institute for Chemical Research, Kyoto University, Kyoto, Japan; 5 Vascular Biology and Molecular Biology, Faculty of Dental Medicine, Hokkaido University, Sapporo, Japan; 6 Department of Materials Engineering, School of Engineering, The University of Tokyo, Bunkyō, Japan; 7 Division of Biological Chemistry and Biologicals, National Institute of Health Sciences, Kawasaki, Japan; 8 Department of Molecular Pathology, Tokyo Medical University, Shinjuku, Japan; 9 Pharmaceuticals and Medical Devices Agency, Chiyoda-ku, Japan; 10 Department of Transfusion Medicine and Cell Therapy, School of Medicine, Fujita Health University, Toyoake, Japan; 11 Department of Transfusion Medicine and Cell Transplantation, Saitama Medical University Hospital, Kawagoe, Japan; 12 Department of Bioengineering, School of Engineering, The University of Tokyo, Bunkyō, Japan; 13 Life Science Research Institute, Kindai University, Higashi-osaka, Japan; 14 Kanazawa Institute of Technology, Nonoichi, Japan; 15 Department of Molecular and Cellular Medicine, Institute of Medical Science, Tokyo Medical University, Shinjuku, Japan

**Keywords:** clinical trials, extracellular vesicles, quality characterization, safety evaluation, therapeutic products

## Abstract

Extracellular vesicles (EVs) serve as an intrinsic system for delivering functional molecules within our body, playing significant roles in diverse physiological phenomena and diseases. Both native and engineered EVs are currently the subject of extensive research as promising therapeutics and drug delivery systems, primarily due to their remarkable attributes, such as targeting capabilities, biocompatibility, and low immunogenicity and mutagenicity. Nevertheless, their clinical application is still a long way off owing to multiple limitations. In this context, the Science Board of the Pharmaceuticals and Medical Devices Agency (PMDA) of Japan has conducted a comprehensive assessment to identify the current issues related to the quality and safety of EV-based therapeutic products. Furthermore, we have presented several examples of the state-of-the-art methodologies employed in EV manufacturing, along with guidelines for critical processes, such as production, purification, characterization, quality evaluation and control, safety assessment, and clinical development and evaluation of EV-based therapeutics. These endeavors aim to facilitate the clinical application of EVs and pave the way for their transformative impact in healthcare.

## Introduction

### What are Extracellular Vesicles (EVs)?

Cells contain and secrete functional molecules such as proteins and nucleic acids in vesicles with lipid bilayer membranes [1]. In the living body, secreted vesicles are transported through body fluids to surrounding cells and cells in distant organs, and they deliver functional molecules to recipient cells. These extracellular vesicles (EVs) are an intrinsic delivery system of functional molecules in our body and have been shown to be involved in various life phenomena and diseases as intercellular communication tools [2]. They have also been reported to be used not only as communication tools but also for discharging unwanted molecules from cells [3]. Such EVs can be broadly classified into two types depending on their formation and secretion route: One is exosome, a vesicle derived from the endosomal system, consisting of intracellular membranous compartments such as early endosomes, recycling endosomes, late endosomes, and the lysosome; the other is a vesicle derived from the plasma membrane called an ectosome. However, confusion often occurred for the following reasons: The definitions of these EVs were vague, it was difficult to identify the formation and secretion pathway unless the moments of release are captured by live imaging technology, and the names were not generally standardized. Under such circumstances, the International Society for Extracellular Vesicles (ISEV) was established in 2011, and ISEV has been defining EVs, standardizing the names, and establishing guidelines, etc. for research. ISEV recommends the use of "EVs" as the generic name of the above-mentioned secreted vesicles [4]. In addition, in 2018, minimal information for studies of extracellular vesicles 2018 (MISEV2018) was published as a guideline, in which it is recommended to call medium/large EVs (m/l EVs) and small EVs (sEVs) according to the size and density of EVs [5]. However, in February 2024, MISEV2023 [1] was published which stated that sEV and exosome are not synonymous and that the size and density of individual vesicles are not always identifiable and depend on the method used to measure size. Therefore, ISEV explicitly recommends not using biogenesis-based terms unless such EV populations can be specifically separated and characterized. Accordingly, in this paper, vesicles with lipid bilayers released from cells are also referred to as EVs.

### Composition of EVs

EVs are limited by a lipid bilayer membrane, which encapsulates the cargo molecules. The cargo of EVs consists of various molecules, and their composition include lipids as well as various proteins and nucleic acids such as microRNA (miRNA) and mRNA. Cells encapsulate these molecules in lipid bilayer membranes of EVs and deliver functional molecules to other cells. EVs delivered to the recipient cells are internalized into the cytoplasm and transported to endosomes. The internalized EVs then release their content of molecules by an indirect route of endosomal escape; however, the detailed molecular mechanism remains unclear. Alternatively, the fusion of EVs with the plasma membrane is known as a direct content of molecules delivery pathway to the recipient cells. As a result of such delivery of functional molecules, the recipient cells show phenotype changes, and gene expression changes in response to them. EVs exert various actions, and the compositions of EVs vary depending on the cell type and even in the same cell, these may vary depending on the surrounding environment [6]. In addition, EVs contain various molecules, including what is called EV markers, such as CD9, CD63, CD81, annexins and heat shock proteins. Such EV markers may be used for identification or quality evaluation of EVs in some cases, but they may not always exist in all EVs [7]. On the other hand, non-EV markers include apolipoproteins, calnexin, Lamin, cytochrome C etc. [1]. As described above, EVs contain a variety of molecules, and thus an individual EV may not always hold the same components. Furthermore, it has also been reported that constituent molecular groups differ depending on the subtypes of EVs [8]. Thus, although EVs are collectively categorized, the component molecules are heterogeneous and it is difficult to define the characteristics of EVs using specific marker molecules. Therefore, EVs are not a term for specific molecules but generally refer to cell-derived complexes consisting of a variety of molecules in lipid membranes, with sizes of several tens of nm to several μm.

### Development of Therapeutic Products Using EVs

Therapeutic products using EVs described here are preparations of cell-derived EVs administered into the body. According to the ISEV position paper [9], EV-based therapeutic products can be classified into at least four distinct categories based on their active components:

i) Native EVs from genetically non-manipulated cells
ii) Native EVs from genetically modified cells without trans-gene products
iii) EVs as drug delivery systems (DDS) loaded with synthesized chemicals or defined recombinant molecules
iv) EVs from genetically modified cells with trans-gene products

EVs in categories i)-iii) are considered biological medicines, while those in category iv) can be classified as gene therapy products that belong to an independent sub-category of biologicals. In this paper, we categorize iii) and iv) as engineered EVs. The characteristics and examples of practical application research for both native and engineered EVs are described below.

#### Characteristics of Native EVs and Examples of Therapeutic Products

EVs exert various effects on cells, tissues, and organs. Among these effects, such as, anti-inflammatory effect, immunoregulatory effect, and tissue repair effect, are expected to be used as therapeutic drugs for specific diseases. In particular, attention has been focused on EVs derived from mesenchymal stromal/stem cells (MSC). MSCs are known to have an ability to support the repair of tissues injured in a variety of ways and are now expected to be a cell source in regenerative medicine [10]. Recent study results indicate that a large part of the therapeutic effect of MSCs is due to secretions of MSCs (secretomes), although in some cases the cells themselves differentiate into the tissue cells to repair the tissue. Secretome includes various EVs, and in fact, therapeutic effects in the use of EVs of MSCs have been frequently reported [11, 12]. Kordelas et al., showed that the use of EVs derived from MSCs led to significant improvement in GvHD symptoms, remarkably reducing the dosage of steroids, albeit in a one-patient result [13]. In recent years, therapeutic effects on humans have also been confirmed. Austrian and German research groups conducted the first-in-human treatment in which EVs of MSCs derived from umbilical cord were applied to the inner ear to attenuate inflammation as an adverse reaction due to cochlear implantation [14]. The results have shown improved speech perception. Furthermore, acoustic trauma causing hearing loss has also been investigated in an experiment in mice, and it has been clarified that administration of EVs of MSCs derived from umbilical cord to the inner ear improved hearing loss and protected hair cells from noise-induced trauma, demonstrating the therapeutic effects in EVs of MSC derived from umbilical cord [15]. In addition, Other clinical trials are underway to investigate the potential usefulness of EVs derived from MSCs as a treatment for COVID-19 [16].

Moreover, the involvement of EVs in immune control has been revealed in early EV studies, and it has been reported that EVs derived from B cells and dendritic cells (DC) were involved in antigen presentation and activation of CD4 positive T cells [17–19]. Based on these reports, a method in which EVs are used in immunotherapy to activate immunity as a vaccine and prophylactic use were proposed. In 2002, a phase I study in melanoma patients in France [20] and a clinical study in patients with non-small cell lung cancer (NSCLC) in the US [21] were conducted. In addition, a phase II study of EVs derived from DCs that have interferon-γ on the surface in patients with advanced NSCLC was conducted in France between 2012 and 2014 [22]. Treatment outcomes appeared not as good as expected, but in some patients, natural killer cells tended to be activated and progression-free survival was prolonged. Currently, clinical studies by other groups are also being conducted, and the development is proceeding toward practical use.

#### Characteristics of Engineered EVs and Examples of Therapeutic Products

In contrast to native EVs, engineered EVs refer to EVs whose contents are changed by genetic modification or transfection to the producer cells, EVs for which a peptide or antibody is conjugated to the surface after secretion from the cells, or EVs in which a specific drug is encapsulated by application of physical force or other methods [23, 24]. The primary purpose of use of engineered EVs is related to the use in DDS because EVs can deliver functional molecules to remote organs and specific tissues and cells. Nano-sized EVs (sEVs) are less immunogenic and less mutagenic than viral carriers [25] and are biocompatible. Thus, EVs are not recognized as foreign substances and can function adequately *in vivo*. Furthermore, nucleic acids in EVs have been reported to be resistant to degrading enzymes, such as nucleolytic enzymes in blood. Thus, a possibility as a stable DDS carrier in circulation has been increasingly expected. In addition, it has been suggested that EVs may have a specific tropism towards target cells, and the construction of a delivery system with high specificity in addition to stability and low toxicity is expected [26]. Therefore, engineered EVs, encapsulating a therapeutic drug or equipping preferable functional features to EVs with the above characteristics, have been prepared and their therapeutic effects have been investigated. In 2011, researchers from Oxford University showed the potential of EVs as a DDS carrier. In this report, DCs were transfected with an expression vector for a rabies virus glycoprotein-derived peptides (RVG peptides), and then EVs were isolated and loaded with siRNA using the electroporation method [27]. This delivery system employs the RVG peptide for specific targeting of nerve cells and includes siRNA as a pharmaceutical drug. Starting with this report, research reports on the application of EVs to DDS have been increasing. However, many studies have used electroporation for loading siRNA into EVs, but some researchers question its reliability. For example, Kooijmans et al. demonstrated that electroporation can cause RNA aggregation and EV instability, resulting in low loading capacity [28]. Therefore, it should be noted that the method of loading siRNA into EVs is controversial. Also from Japan, Tokyo Medical University reported in 2013 on the preparation of EVs displaying GE11 peptide, an artificial ligand of EGFR, loaded on the membrane, targeting tumors expressing EGFR [29]. In addition, there is a report on the therapeutic application of EVs using engineered EVs, where a tumor-suppressor miRNA let-7a was transfected into the producer cells, and EVs containing a large quantity of let-7a were purified. As in the above examples, engineered EVs or DDS preparations are currently obtained by the genetic modification or transfection to the producer cells or by the loading of specific molecules (however, it should be emphasized that efficient loading of target molecules into EVs requires fusogenic components that guide the target molecules to the EVs) or drugs after the purification of EVs (these two methods are combined in some cases). However, general approaches for practical application have yet to be established, with many of the problems being pointed out.

### Problems in Development

There have been many papers and reports from clinical studies on the possibility of EV therapeutic preparations. However, there has been scarce knowledge based on regulatory science for actual manufacturing of products (therapeutic drugs), and various problems have been pointed out and are listed below.

#### Selection of Producer Cells

EVs are biologically active substances derived from cells. However, they are not unique or homogeneous but contain different molecules depending on the producer cells and cell conditions [30]. In addition, there are concerns that the properties of primary cells such as MSCs and other stem cells may differ depending on the donor from which they are derived and that the properties may differ from batch to batch, even with the same donor. As a result, the therapeutic potential of the produced EVs may be different. To avoid such problems, pooling cells obtained from multiple donors and establishing a cell bank is desirable. Thus, in EV therapeutic preparations, quality control of the cells producing EVs, is important from the perspectives of quality control of preparations and obtaining EVs with the intended activity. Establishment of cell culture methods and quality control of culture media are also required [31]. In addition, it will be necessary to decide how to establish rules for the use of genetically modified cells. There are studies using not only cultured cells but also, for example, body fluid- and milk-derived EVs as well as vesicles obtained from plants as the source of EVs for DDS [26], and it is necessary to examine how to deal with cases without culture.

#### Safety

There are several safety issues; however, this review will focus on two of them below. The first is the safety of EVs themselves. The second is related to contamination with, for example, mycoplasma, viruses, endotoxins, and medium components. For the first issue, EVs are involved in various physiological phenomena and diseases, and there are not only EVs that exhibit beneficial efficacy showing the therapeutic effect. For example, EVs derived from cancer cells are known to promote cancer malignancy [32] and may cause unexpected adverse reactions in some cases. Therefore, sufficient consideration is required for development and manufacturing. The second issue includes adverse reactions, etc. caused by substances other than EVs. Because adverse reactions caused by contaminants such as mycoplasma, viruses, endotoxins, and medium components can be substantially prevented by quality control, it is important to establish appropriate control methods.

#### EV Purification Methods

There are many EV purification methods while the best applicable methods have not been established at this time, which has been an issue. Although various purification methods have been suggested, each has its pros and cons, so combining several methods for EV purification would be better. For example, many reports and studies have used the ultracentrifugation methods alone. However, in practical application, the use of ultracentrifugation is considered difficult because multiple samples and large quantities of samples cannot be processed with this method. Moreover, a large quantity of impurities other than EVs remain with some purification methods. Thus, when considering which method should be employed for purification, it is important to evaluate the method based on the purity of EVs so that adverse reactions are also reduced. Furthermore, as there might be a difference in the active components depending on the size of EVs or active component might be contained only in EVs of a specific size, determination of the size may also be important. Therefore, methods that purify only EVs containing active components are more preferable.

#### How to Define the Active Components and Dosage Units

As the cargo of EVs consists of various molecules, not all the molecules are active components. It is desirable to list the components of the preparation, but it is unrealistic to list all components for every lot because of the heterogeneity. Therefore, it is desirable that the active components and the mode of action have been clarified for the formulation, and it is further desirable to determine at least the amounts of these active components. For this purpose, it is useful to establish and use *in vitro* evaluation systems (potency assays) for their efficacy [33]. However, it is not easy to elucidate the mode of action in some cases. In such cases, it is necessary to conduct multifaceted evaluation using multiple *in vitro* evaluation systems, or secure the efficacy using *in vivo* evaluation systems as necessary. In addition, when administered to the body, the unit of EVs to be administered should be clarified. For example, the amount of EV protein, the number of EV particles, or the amounts of active components may be considered. Additionally, it is decided to clarify the conditions, such as which reagents and instruments were used to determine the protein concentration and particle count, and to fix the measurement conditions.

### The Roadmap to Realize EV-Based Therapeutics

**Fig. 1:**
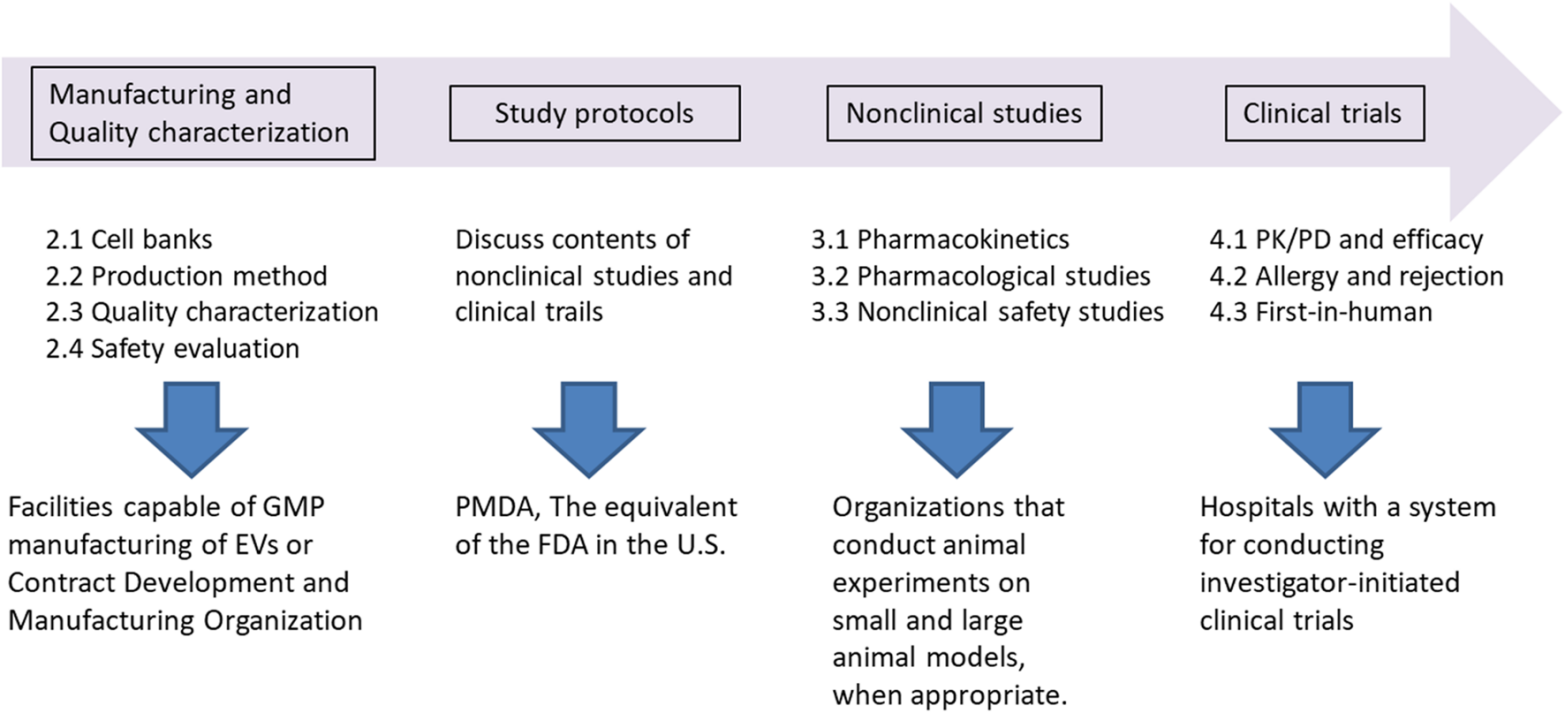
What to do at each step from manufacturing to clinical trials and cooperative partners.

The key steps involved in the development and realization of EV-based therapeutics are illustrated in Fig. 1 and elaborated upon in subsequent sections. Seamless collaboration with partners throughout the entire process of EV manufacturing, characterization, nonclinical studies, and clinical trials is critical for the success.

## Manufacturing Method Development and Quality Characterization

### Establishment and Characterization of Cell Banks

In order to ensure the quality of EV preparations, isolation of cells or establishment of cell lines and their control is necessary, and it is important to maintain various characteristics that may affect the product quality in an appropriate range throughout the period of manufacturing culture. As shown in Table I, human autologous cells, human allogeneic cells such as MSCs, iPS/ES cell-derived cells, and established cell lines are used for manufacturing of EV preparations. Except when autologous cells are used, a cell bank should be established according to The International Council for Harmonisation of Technical Requirements for Pharmaceuticals for Human Use (ICH) Q5D guideline [34] or other appropriate documents and qualified before use in manufacturing. The essential principles when conducting cell culture are outlined in the Guidance document on Good Cell and Tissue Culture Practice 2.0 [35]. The requirements of Good Manufacturing Practice (GMP) apply after establishing cells or cell lines for clinical production to all subsequent processes. When establishing the cell banks, it is critical to confirm the identity of cells by testing the presence of appropriate marker molecules, and comparability tests such as comparative analysis of critical surface marker expressions should be designed to demonstrate identity between the donors, master cell banks and working cell banks.

**Table I:** Characters of EV-Producing Cells

Cell source		Establishment of cell bank for manufacturing	Establishment of cell bank from a single clone	Cell homogeneity*
Primary cell	Autologous	impossible – unnecessary	impossible	impossible
	Allogeneic	possible	difficult	difficult
iPS/ES cell-derived cells		possible	possible	possible
Established cell line		possible	possible	possible

*When relative evaluation is performed based on an established cell line that has already been widely used in the manufacture of therapeutic protein drugs

In addition to Table I, genetically modified cells prepared by transfer of a specific gene, etc. may be used. Characteristics of each cell source, points to consider for establishment of cell lines for manufacturing, and items recommended for establishment, evaluation, and control of cell banks are shown below. For these items, the methods for establishment and control of cell banks used for biopharmaceuticals and cell therapy products can be referenced. Establishment of cell bank is an important step to ensure the reproducibility between the EV production batches under GMP manufacturing.

#### Characteristics of each Cell Source and Points to Consider for Establishment of Cell Lines for Manufacturing

To ensure consistency of cell characteristics throughout the manufacturing culture period, a homogeneous population of cells from cloned cells should be obtained, where possible, so that a cell line for manufacturing is established. In the cases of genetically engineered cells used for manufacturing the engineered EVs, the homogeneity of the cells in terms of the transgene is important. If it is difficult to obtain a homogeneous cell population, it is desirable to identify cells producing EVs that have active components in a heterogeneous cell population to the extent possible, but the method has not been fully established. The history of steps for the isolation of cells and the establishment of cell lines used for the establishment of cell banks should be clearly recorded.

- Human allogeneic cells

It is difficult to obtain a homogenous cell population, and multiple types of cells may be included. Virus safety is an issue because cell banks are established for each donor. In addition, when a cell bank is renewed, assurance of the comparability of the quality of EVs as the final product before and after the renewal will be an issue. The process is identical with the cell therapies and their regulatory requirements can be referred.

b) iPS/ES cell-derived cells

Cloned cells can be used to establish cell lines for manufacturing; however, the phenotype is not always stable, and thus established cell lines and cell banks derived from these cell lines are not necessarily homogeneous cell populations. The risk of carcinogenicity due to contamination with cell-derived DNA or inclusion of EVs derived from tumorigenic cells in the cell bank may need to be considered.

c) Established cell lines

Large scale manufacturing is possible, and the method established in biopharmaceuticals can be referenced for manufacturing method development. Because cell lines for manufacturing can be established from cloned cells, it is expected that cell banks will be established using homogeneous cell populations. Immortalization methods includes spontaneous, viral-induced, cancer-derived, and telomerase-induced approaches. Among them, telomerase immortalized cells are considered to maintain primary cell functionality well [36], and a recent report has shown feasibility in culturing hTERT-immortalized MSCs up to five liters for EV manufacturing [37]. Although there is no definitive evidence suggesting EVs from immortalized cells carrying oncogenic genes (e.g., MYC or E7) have oncogenic potential, theoretical risks cannot be dismissed.

It should be noted that there is no information on the stability of the phenotype during long-term repeated subculturing when a new cell line is established. The risk of carcinogenicity due to contamination with cell-derived DNA or inclusion of EVs derived from tumorigenic cells in the cell bank may need to be considered. As a possible evaluation method, testing the behavior of primary cells those were exposed with EVs from immortalized cells can be considered. However, the test method to evaluate carcinogenicity has not been established and knowledge of this points has not been accumulated yet. To enable the clinical application of EV products produced from immortalized cells with such concerns, establishment of reliable method is important.

For genetically modified cells, analysis of the gene expression construct and control of the cell bank should be performed with reference to the ICH Q5B guideline [38] or other appropriate documents in addition to the above points to consider.

#### Establishment of Cell Banks

If human allogeneic cells are used, a donor cell bank (DCB) shall be established using cells prepared from raw materials collected from a qualified donor. For iPS/ES cell-derived cells, established cell lines, and genetically modified cells, a master cell bank (MCB) shall be constructed from established cells, and a working cell bank (WCB) shall be constructed using a part of MCB. The routine manufacturing shall be performed using a WCB. If the WCB needs to be replenished, the cells from the MCB shall be prepared again for renewal of the WCB.

The established cell banks should be qualified through characterization, purity, and storage stability tests as shown in Table II. In addition to these, evaluation of the gene expression construct is required when genetically modified cells are used. Furthermore, to evaluate the stability throughout the manufacturing culture period, the same tests shall be conducted also for the cells at the limit of *in vitro* cell age (LIVCA) for manufacturing of EVs.

**Table II:** Qualification of Cell Banks

	Characterization test	Purity test	Viral safety test	Storage stability test	Gene expression construct*
DCB	√	√	√	(√)	-
MCB	√	√	√	√	√
WCB	-	-	-	√	-
LIVCA	√	√	√	-	√

*For genetically modified cells

#### Characterization Test of Cell Banks

The characteristics of cells and produced EVs shall be evaluated. Regarding the characteristics of cells, to confirm identity of the cells (the cells are the intended ones, phenotypes such as genotype, isozyme, and expression of cell surface marker molecules) shall be analyzed. Demonstration of identity of the cells is critical in production. Regarding the characteristics of produced EVs, analysis shall be performed on items appropriate for evaluation of cells for manufacturing among the quality characteristics whose variations may affect the efficacy/safety (i.e., critical quality attributes) with reference to "EV-specific quality characterization" in the Section "EV-Specific Quality Characterization" of this article to confirm that EVs with intended characteristics are produced. MSCs, in particular, are known to have different characteristics depending on the tissue of origin, so necessary characterization, including the confirmation of appropriate cell surface markers, should be performed. For engineered EVs, confirm that the intended molecule is included in the EVs after modification.

#### Safety Testing of Cell Banks

Because EV preparations have similar physicochemical properties to those of viruses such as the particle size and charge, removal and inactivation of viruses in the purification process are assumed to be difficult. Thus, for cells used for manufacturing, sufficient virus safety analysis should be performed with reference to the ICH Q5A guideline [39]. Cells in which a type of endogenous virus known to be an obvious hazard or such virus-like particles are detected by a retrovirus test or *in vitro* test should not be used for the manufacturing of EV preparations. For prevention of contamination with adventitious viruses, etc. during the manufacturing process and removal/inactivation of viruses during the purification process, see Section "Safety Evaluation Of Infectious Agents Such as Viruses Contaminated In EVs – Measures Taken by Overviewing Manufacturing Processes" of this article. It is also necessary to confirm that contamination with microorganisms such as fungi and mycoplasma does not exist. Endotoxin is another routine test required.

#### Analysis of Gene Expression Construct

It is required when genetically modified cells are used. The nucleic acid sequence of the vector used for gene transfer shall be determined with reference to the ICH Q5B guideline or other appropriate documents, and the entire nucleic acid sequence of the target gene introduced in the established cell and the copy number of transferred genes shall be analyzed. It should be confirmed that there is no insertion or deletion in the transferred target genes.

#### Analysis of Cells at the Limit of In Vitro Cell Age (LIVCA)

In addition to the analysis of the MCB, WCB, or DCB, main characteristics of the cells at an *in vitro* cell age reached at the end of the longest culture period (or longer) for EV manufacturing shall be determined focusing on the characteristics of the produced EVs in order to confirm that the required cell characteristics are maintained throughout the manufacturing culture period. When genetically modified cells are used, it is necessary to confirm the sequence and copy number of the transferred genes, etc.

#### Storage Stability Test of Cell Banks

Because cell banks are stored frozen, stability should be checked periodically. The endpoints of the storage stability test should follow those of the characterization tests conducted at the establishment of cell banks.

#### Renewal of Cell Banks

The MCB and WCB are permanently stored throughout the product life cycle, but they need to be renewed depending on the number of vials used for manufacturing. If renewal is expected, the method and criteria for renewal should be specified in advance. Because the DCB is repetitively prepared from a new donor, it should be prepared in accordance with the predetermined preparation method of the DCB and the conformity to the criteria should be confirmed. In both cases, the criteria for renewal should be set based on the results of characterization (e.g., identity test) and purity tests for microbiological contamination performed at the establishment of the cell bank. When a cell bank is renewed, evaluation of virus safety is essential.

### Manufacturing Methods of EV Preparations

Basically, cell culture methods for the manufacturing of EV preparations shall use cell sources as stated in Section "Establishment and Characterization of Cell Banks". Because the components of EVs secreted may change depending on the culture conditions as well, it is important to use a consistent culture method and prevent contamination with factors from external environments such as viruses. It is a prerequisite for EV therapeutic drugs that EVs themselves that are secreted from cells under such conditions exert the intended pharmacological action. This means that, for preparations made from the culture supernatant without the EV purification step, substances other than EVs, for example, cytokines not derived from EVs, may also be pharmacologically-active, but such preparations are not categorized as EV preparations. Thus, it is desirable to purify EVs if it is possible, but it is difficult to completely separate EVs from other substances with the current technology [40]. Some molecules co-isolated with EVs, such as proteins in blood plasma, may not be considered 'contaminants' but rather integral components of a bioactive EV 'corona' that are desirable in the final product [41].

On the basis of this matter, points to consider in the process of purification/recovery and formulation of EVs as the active factor from culture medium are shown below.

#### Culture Methods

The quality characteristics of the EVs are considered to be affected by the conditions and status of the cultured cells and the culture conditions. Thus, sufficient characterization of the cells, establishment of the manufacturing process to produce EVs with consistency, and process control in manufacturing are very important. Furthermore, precautions specific to EVs are described below.

Secretion of EVs from cells are affected by the medium components, culture conditions, and the conditions of cells (5), and thus process regulate to control the quality characteristics for the target EVs is also important. For example, apoptotic vesicles have different functions and roles from other EVs (exosomes and microvesicles) (2). Considering apoptotic bodies may exert different effects, it is important to monitor apoptotic cell rates in culture. Moreover, as described above, it is assumed that the components of EVs may vary depending on the conditions and characteristics of the cells. It is important to control the composition of culture media containing additives such as fetal bovine serum (FBS) and cytokines, culture temperature, oxygen and CO_2_ concentrations. In particular, evaluation of the safety of raw materials such as factors added to culture media and their residual in the final product is required. When utilizing biological origin raw materials such as FBS containing animal-derived components and human platelet lysate (hPL), which has been increasingly adopted as a substitute for FBS, it is essential to conduct evaluations, including assessing the risk of infectious agents. A key distinction between FBS and hPL lies in their xenogeneic or allogeneic origins. Despite hPL being considered safer than FBS, there remains a potential risk of pathogen transmission from human-derived products. It is also necessary to evaluate whether these components are sufficiently removed/reduced in the purification process. Therefore, except in exceptional cases, it is recommended that FBS be avoided in GMP production of EVs. In addition, because cell density also affects the amount and components of EVs, it is important to evaluate the equivalence before and after the change in manufacturing scale-up, etc. Thus, it is recommended to establish several checkpoints from culture to EV purification. This means that it is advisable to confirm before purification whether certain criteria are satisfied, such as the conditions of cells after seeding (morphology, apoptotic cell rate, total cell number, and survival rate), amount of EVs present in the culture medium before EV purification, and the amounts of active components contained in the EVs. During the process development, it is important to evaluate the consistency of the EV characteristics in terms of composition and phenotype at the different harvest times that are expected to be used in commercial manufacturing batches. It is desirable to evaluate the characteristics of EVs harvested from the cell culture supernatant beyond maximum culture period that will be used in commercial production, and confirm their consistency. It is important to ensure the defined culture period does not have an impact on quality of EVs. In addition, the details of the points to consider concerning inclusion of impurities from the external environment, especially infectious factors such as viruses, are described in Section "Safety Evaluation Of Infectious Agents Such as Viruses Contaminated In EVs – Measures Taken by Overviewing Manufacturing Processes".

#### EV Purification Methods

Although various EV purification methods have been proposed, it is necessary to develop methods in consideration from the purification process at the pilot scale in the early stage of development to the commercial manufacturing after approval. At the early stage of development, the characteristics of the active component may not be fully understood, and the purification method may not have been established. However, it is desirable to have the purification method established before producing EV preparations (GMP-grade production of EVs) to be used in nonclinical studies. This means that it is required to clarify the component necessary for its biological activity (efficacy) and establish a purification method of EVs containing the active component. In addition, it is necessary to establish purification processes that can reduce inclusion of unintended EVs and culture medium components. Furthermore, because commercial preparations are expected to be administered to a large number of patients, it is necessary to produce them at a scale applicable to commercial preparations. Thus, it is desirable to use column chromatography or other methods that can be scaled up as the culture scale is increased. For example, purification of EVs by using the size exclusion chromatography method, which sieves molecules on the basis of size, and the anion exchange chromatography method, which separates molecules on the basis of physicochemical properties such as electric charge, and the separation results have already been reported [42, 43]. However, it is not necessary to limit the method to column chromatography, and the purification method shall be selected according to the characteristics of EVs with the intended biological activity. Moreover, as discussed in Section "EV Purification Methods", several EV purification methods can be combined to accommodate scale-up. In any case, the important point is to consistently use the same method of purification without changing the purification method in the process. Examples of purification methods using individual characteristics and points to consider are described below. In addition, in any purification method, the application of ultra-concentration, etc. should be considered for bulk harvest recovered at the end of the culture process, as necessary. Furthermore, in any purification step, it is necessary to establish a manufacturing method with indices such as removal of process-derived impurities (e.g., ligands and column carriers derived from culture process or used in purification process) and unintended related substances, and in some cases, it may also be considered that the indices shall be the extent of the removal of impurities by comparing before and after purification.

Purification by the ultracentrifugation method is often performed in research stages and in laboratories, but the application to commercial preparations is difficult as described above, and concentration of bulk harvest is required as a pretreatment. Similar to ultracentrifugation, there are methods to precipitate and concentrate EVs by low-speed centrifugation using commercially available polymers such as PEG as EV purification reagents in research stages and in laboratories, but there is a concern that the reagents may remain and will concentrate even many free proteins when these methods are used [44, 45]. Thus, it is essential to establish a method to determine the residual amounts of impurities after purification.

In cases where the active component is a protein on EV membranes, affinity purification using antibodies, etc. is a useful method for purification by using those as indices. Furthermore, affinity purification of CD63 and CD9, which are referred to as EV markers, is also possible. However, these markers do not necessarily exist in all EVs [7], and it is necessary to determine whether or not the purified CD63 or CD9-positive EVs have the intended biological activity also from the viewpoint of the heterogeneity of molecules encapsulated in EVs. In addition, the safety of reagents such as buffers used to remove EVs from the antibodies and for purification should also be confirmed in consideration of potential inclusion of impurities. If necessary, solvents shall be replaced further by another method.

Purification based on size is also useful when EVs with the intended biological activity are defined with their size, for example, in cases where small particles with a size of ≤ 200 nm (sEVs) strongly exhibit the intended biological activity, or conversely, large particles (m/l EVs) ≥ 200 nm have the activity. The examples include separation by size exclusion chromatography and filtration using ultrafiltration membrane. However, in these methods, when proteins aggregate or other materials of the same size are present, the amounts of remaining impurities may increase, and thus it is necessary to establish a method to determine the residual amounts of impurities.

### EV-Specific Quality Characterization

Because EV preparations are considered to be a type of biopharmaceutical produced from cells, the points to consider described in "Specifications: Test Procedures and Acceptance Criteria for Biotechnological/Biological Products" (ICH Q6B) and in "Pharmaceutical Development" (ICH Q8) are useful for the quality characterization.

In addition, the quality characterization needs to take into account the characteristics specific to EVs. After full consideration of the development of manufacturing methods in which EVs with the intended biological activity and active components are purified as much as possible or to a certain level, the characteristics of the EVs obtained by the purification method shall be clarified by EV-specific characterization. However, because the separation/purification methods of EVs have not yet been fully established, it is necessary to pay sufficient attention to inclusion of components other than intended EVs that remain after purification. Consideration should also be given to the stability of EVs (potential degradation and transformation of products) during the manufacturing process and storage period. Qualitative and quantitative detection, establishment of a contamination acceptance limit, and safety evaluation are required for potential impurities, degradants, etc.

#### EV-Specific Characterization

EV-specific characterization includes analyses of EV composition, physicochemical properties, and biological activity. Because EVs consist of heterogeneous particle populations, evaluation of the distribution and variability of characteristic values for each particle is considered important in addition to the evaluation of the overall mean characteristic values of particles. An approach to evaluate EVs at single particle-level resolution is considered useful for understanding the quality characteristics of EV preparations, and thus it is desirable that such analytical methods are incorporated in test items. In the field of EV research, development of new purification and analytical technologies and improvement of existing technologies are being carried out every day, and thus they should be incorporated in a timely manner. Major methods used to evaluate EV characteristics are shown in Table III, and are described in detail in the following subsections.

**Table III:** Characterization of EVs

EV character	Analytical method
a) Composition (proteins, nucleic acids, lipids, metabolites, sugar chain modifications)	Mass spectroscopy Western blot DNA/RNA sequencing Microarray Flowcytometry RT-qPCR HPLC ELISA
b) Physicochemical properties (particle size/number, zeta potential)	Nanoparticle tracking analysis Dynamic light scattering Tunable resistive pulse sensing Electron microscopy Atomic force microscopy Fluorescence correlation spectroscopy Electrophoretic light scattering
c) Biological activity	Potency assays to evaluate or predict the efficacy of EV therapeutics (e.g., quantitative measurement of active components and enzyme activity; assessment of pharmacological activity in cells, tissues, and animals)

- Composition analysis

Analyses related to molecular composition, positive rate of EV marker molecules, and surface molecular profile are included. Determination of the presence or absence and quantitative analysis of EV-related molecules such as proteins, lipids, sugar chains, RNA, and metabolites present on the surface and inside of EVs are performed. Quantitative analysis should be a priority when possible. Moreover, when the target active components have been identified or when engineered EVs are used, qualitative and quantitative analysis of the target components and the molecules transferred by modification is necessary. Tetraspanins (e.g., CD9, CD63, and CD81) and late endosome-associated proteins (e.g., Tsg101 and Alix) are commonly known as EV marker molecules. Other examples include the use of ganglioside GM1 as a marker for endosome-derived EVs and the use of major histocompatibility complex (MHC) class I as a criterion for dosage of DC-derived EVs. In any case, it is important to analyze with multiple combinations of EV marker molecules that are suitable for the purpose, keeping in mind that there are currently no EV marker molecules for which a consensus is reached. In fact, an ISEV position paper [9] stated that at least three types of proteins specific to EV fractions or other EV-associated molecules should be analyzed. However, MISEV is primarily focused on research, monitoring a wide array of markers that are not directly relevant to therapeutic applications. Although MISEV guidelines can provide valuable insights into EV preparations, compliance with MISEV criteria is not required unless it is directly relevant to the product. In addition, it is highly recommended to use orthogonal approach for composition analysis and monitor the multiple parameters from different analyses. MISEV2023 also stated the need to evaluate the topology of the particles, which is to confirm whether the constituents of EVs are present in the interior of particles or on the surface of particles, considering that EVs may be decomposed/changed in the manufacturing /storage steps [1]. On the other hand, there is a possibility that components not related to EVs (or components that are considered less relevant) may be included in EV preparations as impurities. The examples include factors related to intracellular organelles such as mitochondria and Golgi bodies, culture medium-derived components such as serum proteins, and virus particles and their fragments. For impurities that may be included, their detection methods and assay methods should also be investigated. For EV composition analysis, bulk analytical methods such as Western blotting and mass spectrometry are commonly used. Meanwhile, the flow cytometry method has been developed intensively in recent years, which is expected to enable various molecular composition analyses at a single particle level.

b) Analysis of physicochemical properties

The examples include the analysis of particle size, particle number, and surface charge (zeta potential). High-resolution imaging using microscopy such as electron microscopy and atomic force microscopy can measure particle size by direct observation, but it is practically difficult to obtain a statistically sufficient amount of data. Dynamic light scattering (DLS) is an established method widely used for evaluation of particle size distribution of microparticles in a sub-micron region, but it should be noted that the accuracy of measurement may be deteriorated for polydisperse particles. Single particle analysis methods are considered to be preferred for the analysis of the size and number of heterogeneous microparticles, and nanoparticle tracking analysis (NTA), tunable resistive pulse sensing, fluorescence correlation spectroscopy (FCS), etc. are often used. It should be noted that the measured values of particle size distribution are readily affected by the measurement method (principle) and equipment (model). For the determination of zeta potential, the electrophoretic light scattering (ELS) method, a combination of the light scattering and electrophoresis methods, has been frequently used. Recently, single particle-level analytical instruments for zeta potential measurement are being developed by improving NTA and the resistance pulse sensing technique. In any analysis, it is required that an individual particle is in a dispersed state, not aggregated.

c) Evaluation of biological activity and related molecules

The biological properties of EVs shall be clarified by the potency assay [33]. Potency assay for EV therapeutics is the assay that is used to evaluate or predict the efficacy of EVs and EV preparations. This includes test methods to measure cellular functions, responses, and other biological activities in the multiple test systems, depending on the characterization of EVs and EV preparations. Functional assay is included in potency assay but should reflect the clinical mode of action of the drug. However, because of their characteristics, it is often difficult to fully elucidate the mode of action of EVs. Thus, it is desirable to investigate the mode of action of EVs as much as possible, and then to set up multiple tests based on the pharmacological action of the EVs with appropriate positive and negative references to clarify the biological activity characteristics multidimensionally. The potency assay is performed using test systems such as cells, tissues, and individual animals in addition to *in vitro* biochemical tests. For example, *in vitro* assays include quantitative measurement of EV active components (e.g., miRNA, mRNA, and proteins) and enzyme activity assays. Furthermore, cell assays include evaluation of cell activity in terms of cell proliferation, migration, toxicity, activation/suppression of immune cells, gene expression regulation, signal transduction, etc. It is considered that the obtained activity values may be normalized by particle number or protein amount depending on the purpose to evaluate the specific activity. Biological activity evaluation of EVs is important not only for clarifying EV properties but also for quality control and securing consistent characteristics of EVs.

#### Impurities in EV Preparations

EV preparations are expected to contain not only EVs with the intended active components but also EV-related substances such as EVs without the active components (unintended EVs) and EVs changed or decomposed during the manufacturing /storage steps (EV variants). It is desirable to eliminate unintended EVs and variants as much as possible by chromatography or purification processes focusing on characteristics such as surface molecules and surface charge. In addition, in the manufacturing of EV preparations, it is considered that impurities such as other EVs, viruses/virus-like particles/microorganisms/mycoplasma, and medium components/reagents may be included via cell substrates, cell culture media, equipment and reagents used for purification and concentration, etc. [46], although some impurities may not be just contaminants but rather contribute to the biological effects and the physicochemical properties of EVs, such as protein coronas surrounding the surface of EVs. Furthermore, there is a sufficient possibility for inclusion of airborne particles in the work environment and artificial microparticles derived from labware such as tubes, particularly during nonclinical production. It should be noted that separation of particles with a similar size to that of EVs is generally difficult once they are included, whether they are of biological origin or artificial substances. Thus, it is extremely important to design and control appropriate manufacturing processes to minimize the inclusion of components other than the intended EVs. In addition, it is important to establish qualitative and quantitative test methods for impurities that may be included and to examine the acceptable limit of impurities from the viewpoint of efficacy and safety. Indicators for evaluation of inclusion of components other than the intended EVs include the evaluation of the proportion of target EV particles by vesicle profile analysis, potency measurement of EV preparations by biological tests, and comparison based on the potency standardized by the number of particles or other quantities.

In the manufacturing of EV preparations, it is desirable to avoid the use of animal-derived raw materials (including humans) to the extent possible. For additives of human or animal origin, viral contamination may occur, and if FBS is used, EVs of bovine serum origin may be included, leading to unexpected biological activities. Generally, use of reagents and recombinant proteins free from animal-derived components is recommended. Also, it is recommended to use media with defined composition for EVs production, which allows a better control of EVs purity, contaminations identification, and batch to batch reproducibility.

#### Stability Study

Given the membrane structure inherent in EV products, it is imperative to meticulously examine and establish formulation and storage conditions. Stability studies represent a crucial aspect of EV quality control strategies. Real-time/real-temperature stability studies are fundamental for defining storage conditions and shelf-life of EV products. During product development, accelerated and/or stress stability studies are also valuable for optimizing manufacturing conditions and formulations. To effectively assess stability, it is essential that established quality attributes and their corresponding analytical procedures consider the critical impact on safety and efficacy of each attribute.

### Safety Evaluation of Infectious Agents such as Viruses Contaminated in EVs – Measures Taken by Overviewing Manufacturing Processes

Because EVs and viruses are similar in size and EVs are highly likely to show the same behavior as that of viruses in processes such as purification because of the characteristics, it is difficult to apply viral clearance processes in manufacturing for biotechnology products [47, 48]. Thus, with regard to virus safety, it is most important to use cells not contaminated with viruses as the manufacturing substrate of EVs, and thus it is important to confirm the eligibility by performing viral screening for donors of cells used for manufacturing of EVs. The collected cells are examined for multi-dimensional infectious factors including viruses and a cell bank (see Section "Establishment and Characterization of Cell Banks") is established by separation and proliferation of the target cells from the collected cells/tissues. Meanwhile, in a case where the patient and the donor are the same person (so called autologous product), the donor screening is not required on the prerequisite that adequate measures are taken against cross-contamination in the manufacturing process.

In the manufacturing of EVs, for example, when raw materials of biological origin are used for cultivation of manufacturing cells, it is important to use materials not contaminated with viruses, etc. In addition, it is important to take measures against contamination with infectious factors such as viruses due to improper handling in manufacturing.

Adventitious virus testing should be performed for bulk harvest produced in a culture process. Drug substance and drug products of EVs are prepared from the bulk harvest, and the application of viral clearance process is often assumed to be difficult in EV purification processes; thus, virus testing for harvest or suitable intermediates after subsequent processes from the bulk harvest is very important as process control. Particularly in cases where a process of EVs concentration is performed, because if there is viral contamination, the viruses are also likely to be concentrated. Thus, in some cases, it may be suitable to perform virus tests on in-process products after concentration instead of bulk harvest.

It has been assumed that among viruses that may be included in EVs, enveloped viruses, which have lipid membranes, are likely to behave similarly because of the similarities in characteristics. Meanwhile, some non-enveloped viruses have been reported to have an envelope when released from cells, and thus careful consideration should be given to these points in testing of bulk harvest, etc. [49].

## Nonclinical Studies

### Pharmacokinetics

In usual drug discovery, absorption, distribution, metabolism, and excretion (ADME) studies are conducted to clarify the pharmacokinetics, but for EV preparations, it is considered difficult to perform any evaluations such as the determination of blood concentrations, absorption or excretion rate for reasons that prepared EVs are not homogeneous, and evaluation methods for separating and analyzing specific EVs containing the bioactive components have not been established at present. Thus, there are technical limitations in the evaluation of pharmacokinetics of EV preparations. In addition, native EV preparations derived from human cells are considered to consist of human-derived components, and thus it may be supposed that there is little need to evaluate the metabolism and excretion. On the other hand, for engineered EV preparations that contain chemical substances, quantitative evaluation of ADME according to the level of exposure to the relevant substances is important. It is considered that biodistribution of EV preparations can be evaluated by the following methods according to past reports, and it may become important information in elucidating the pharmacological and toxicological target organs, duration of action, and mode of action of the relevant EV preparations. It should be noted, however, that artificially-labelled EV preparations may not reflect physiological dynamics by any visualization method.

#### Differences in Biodistribution by Types of EV Preparations

There are over 30 reports on biodistribution using sEVs (Exosome-dominant EVs), mainly by i.v. administration in mice. *In situ* analysis showed that sEV accumulations are observed in the liver, lung, bladder, and spleen in order of strength after 12 h, and the signals tended to weaken except in the lung at 24 h later. Noteworthy are the weak signals in kidney, brain, and heart observed at 1 h after sEV transfer. In ex vivo observation after sEV administration, 12–24 h later, strong signals are observed in the liver, spleen, lung, bladder, kidney, gastrointestinal tract, and bone in addition to the weak signals in the brain, heart, muscle, and pancreas. On the other hand, there are only three reports regarding lEVs (Microvesicle-dominant EVs), strong accumulation in the liver is observed for over 24 h, and like sEV, distributions in lung, kidney, and spleen are also observed. Biodistribution by i.v. administration of the ultracentrifuged EVs, including the above-mentioned sEVs, is strongly observed in the liver, spleen, lung, and kidney regardless of the staining method. Differences in types of EVs, staining method, route of administration, dose, and parental cells appear to be associated with the fluctuation of resulting EV biodistribution to the heart, brain, gastrointestinal tract, bone, pancreas, bladder, and muscle [50, 51].

The biodistribution of EVs differ dramatically depending on the route of administration. Human MSC EVs administered i.v. accumulate in the liver, lung, and spleen within 24 h, in contrast, signals limited to the lung and brain are observed with intratracheal and intranasal administration, respectively [52]. In a study using non-human primate *Macaca nemestrina*, no accumulation is observed in systemic organs after intranasal administration, but strong accumulation of i.v. administered EVs, similar to the murine studies, is observed in liver, spleen, kidney, and lung in addition to the weak signals in the medulla of the brain. This report also reveals that there is an optimal concentration of EVs for long-term persistence of i.v. administered EVs in the blood, and that B cells are mainly involved in their clearance from the blood [53].

Most of the above reports on EV biodistribution have used EV labeling techniques, especially membrane-labeled (amphiphilic) dyes such as PKH and Dil, which are prone to labeling artifacts [53, 54]. In addition, the significant accumulation in the liver, lung, and spleen are due to the long-term retention of the dyes and radio isotopes bound to EVs after macrophage-mediated phagocytosis, and the free dyes and radio isotopes coexisting in the stained EV preparations [54, 55]. The selection of the certain EV populations and the optimization of labeling methods may enable delivery of EVs to target organs. Recently, it has been reported that sEVs derived from adipose mesenchymal stem cells labeled with ultrasmall superparamagnetic iron oxide nanoparticles (USPIO) accumulate in axillary lymph nodes, but not in lung, when administered i.v. to experimental autoimmune encephalomyelitis mice [56]. In PK studies of EVs that affect immunity, it may be necessary to focus on their distribution in lymph nodes rather than in the spleen where dyes accumulate nonspecifically.

#### EV-Labeling and Detection Methods

**Fig. 2:**
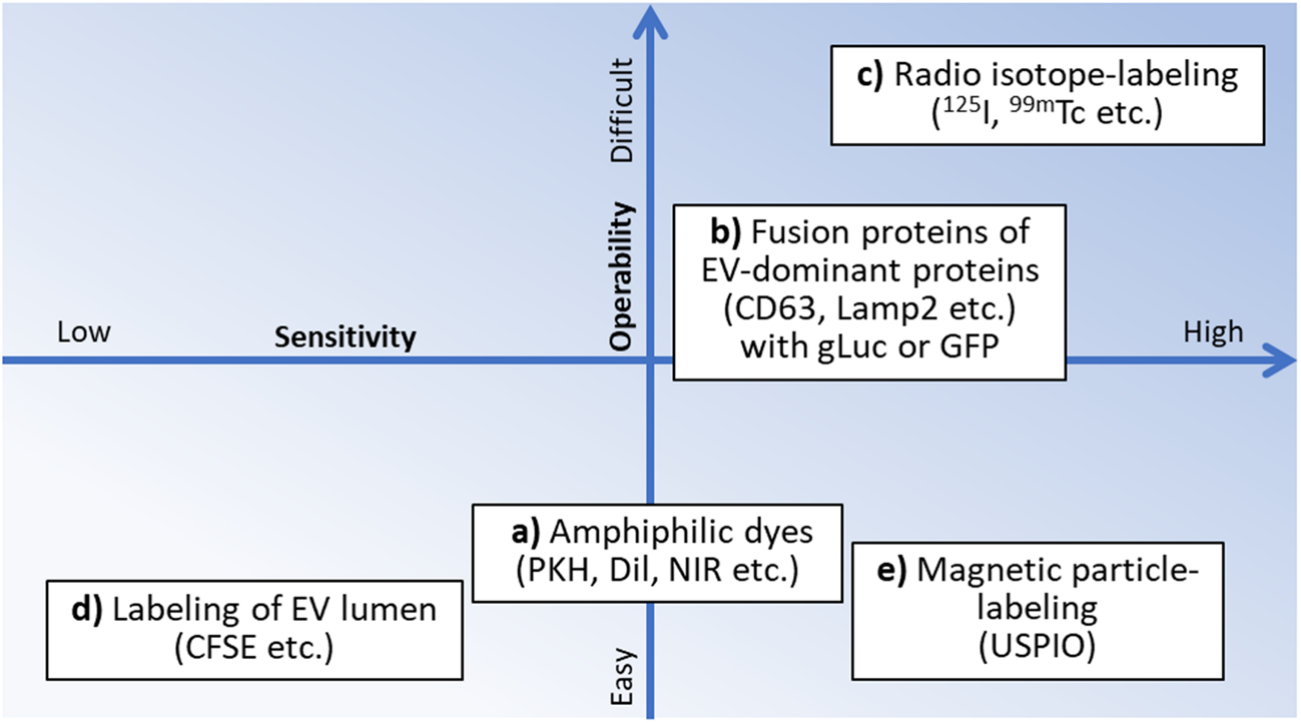
Operability and sensitivity of current EV-labeling methods.

Current EV-labeling methods used for biodistribution studies include the followings, and classified by operability and sensitivity in Fig. 2. If safety issues are observed in the PK test using rodents (mice), other labeling methods, further removal of free dyes (3.1.3) or EVs with higher purity or specificity should be used. For detection at organ levels, IVIS is commonly used for methods a), b), and c). BLI is used for b).　PET and SPECT/CT are used for c). MRI is used for e). In many reports, histological staining of tissues and flow cytometric analysis of tissue-derived cells are performed in PK studies using fluorescent dyes in addition to studies at organ levels.

- EV-labeling using amphiphilic dyes

EV membrane labeling methods using fat-soluble dyes such as PKH dyes, Dil dyes, and near-infrared absorption dyes (NIR) including DiR and DiD are available [57]. These dyes are lipid-like molecular probes, which are inserted into the lipid bilayer membrane, and are used as simple labeling methods in many PK studies, but these dyes are known to deform the EV size and alter their uptake and biodistribution [58]. It will be important to examine *in vitro* whether the target cell specificity of the original EV preparation is maintained in labeled EV preparation. In addition, it should be noted that if lipophilic proteins such as lipoproteins coexist in prepared EV preparations, they will also be stained, leading to erroneous results. For example, as described in 3.1.1., the accumulation of large amounts of EVs in the liver, which has been observed in biodistribution studies of EV preparations using this labeling method, is considered to be one of the erroneous results. However, because this labeling method is the simplest, it is recommended for exploratory investigations at the organs/cell levels especially in the early stage of development.

b) Labeling using EV-dominant proteins

Labeling methods using fusion proteins of reporter protein such as gLuc and GFP, in conjunction with CD63 and Lamp2, which are concentrated in EVs, or lactadherin, which has an affinity for EVs, or Gag, which binds to PI(4,5)P2 and is abundantly present in the inner leaflet of EV lipid bilayer [59, 60]. It is considered that these labeling methods are more sensitive and quantitative than the labeling methods shown in a) and are appropriate for *in vivo* imaging. However, because the amounts of EV proteins used are not the same among individual EV particles, the obtained results may be biased. There is also concern that these fusion proteins prepared by genetic manipulation may affect on the biological characteristics of EVs. Thus, although these labeling methods are highly sensitive, it is recommended that they should be used in combination with the labeling method a), c), d), or e) to assess biodistribution at organ levels.

c) Labeling using radio isotopes

There are labeling methods using a fusion protein of lactadherin-streptavidin with radioisotopes such as biotinylated ^125^I or ^99m^Tc [61]. These labeling methods are the most sensitive reported to date and suitable for quantitative *in vivo* imaging, and the results are unlikely to be biased. However, these methods require operations in RI facilities where animal experiments can be conducted. Thus, these methods are recommended in investigation of biodistribution of EVs at low doses, but the method a), b), or e) should be used for investigation at medium or high doses. Consistent with b), this staining method is also biased, so it is important to also perform in combination with a), d), or e).

d) Fluorescent staining of EV lumen

There are fluorescence staining methods for EV lumen using SytoRNASelect, CFSE, etc. SytoRNASelect and CFSE. These are the membrane permeable dyes, and may be the most suitable dyes to study unbiased biokinetics because their fluorescence is retained in the EV lumen. However, in addition to the cytotoxic property of CFSE, sufficient sensitivity may not be obtained because of the weak fluorescence at present. Because these methods are mainly used in studies of microvesicles (lEVs), without protein markers, the methods in a) to c) should be used in PK investigations of exosomes (sEVs). They are recommended for tissue staining in studies of biodistribution.

e) Labeling using magnetic particles

EVs bound with magnetic particles (Fe3O4: USPIO) with an average diameter of 5 nm have been administered to mice [56]. USPIO-EVs may be used more frequently in the future PK investigations because of its characteristics of non-invasive, highly sensitive, and has deep penetration.

#### Removal Methods of Free Fluorescent Substances and Radio Isotopes

To understand the accurate biodistribution of EVs, free fluorescent substances and radio isotopes remaining after the staining procedure must be removed by ultracentrifugation, density gradient method, ultrafiltration, or size exclusion chromatography including spin column [54]. To examine biodistribution, a large amount of EV is required, and density gradient method may not be suitable. For ultrafiltration methods, the pore size (MWCO) at which EVs remain completely must be considered. It has been reported that there is no EV loss from the culture supernatant with 750 kDa MWCO (approximately 50 nm in pore diameter) ultrafiltration [43]. Since low-density lipoprotein (LDL) and very-LDL (VLDL) is stained by lipophilic dyes such as PKH, Dil, etc., there are limits to the removal of free dyes using ultracentrifugation [62, 63].

#### Scientific Interpretation of Uptake in Organs and Cells

As mentioned in 3.1.1, EV organ-level distribution varies under different conditions [50, 51]. Understanding the irregular distribution of administered EVs between target and non-target cells and organs is crucial for assessing human risk. To assess this distribution effectively, conducting PK studies at the organ and cellular levels using various labeling techniques and administration methods for targeted EVs is recommended. Additionally, comparative PK studies between engineered EVs and their counterparts from original cells pre-modification are essential for comprehending aberrant distribution patterns.

#### Countermeasures for Reducing Distribution in Non-Target Organs and Cells

In addition to reconsidering the EV staining method, it is very important to remove free dyes as much as possible, as mentioned in 3.1.3. As described in 2.3.2, EV preparations contain EVs with the intended active components, unintended EVs, EV-related proteins, viruses, mycoplasmas, culture medium components, plastic components, reagent components, etc. If the EV population of the EV preparations has been identified, the EV population should be isolated and purified as much as possible. If the prepared EV contains a large amount of components other than EV, such as unnecessary proteins and lipids, the EVs should be prepared by methods to achieve high purity (for example, the affinity method [64] and ion exchange method [43]). For engineered EVs, enhancement of specificity (affinity) to target cells would be effective.

### Pharmacological Studies

Pharmacological studies using EV preparations are basically the same as studies conducted for low-molecular drugs or protein preparations and studies that are suitable for individual diseases shall be conducted using cells, tissues, individual animals, etc. However, for the selection of the control group and dose unit of EVs, the following measures may be considered as EV-specific items. Furthermore, as most of the active components of EVs are proteins and nucleic acids, attention should be paid to differences in animal species in pharmacological studies.

#### Control Groups

The MISEV2023 recommendations for functional studies of EVs emphasize conducting physiologically informed dose–response and time-course studies [1]. Carefully selected EV negative controls should be employed to account for background EV activity and non-specific effects. For instance, utilizing unconditioned medium or EVs from unrelated cell types as controls may be appropriate. Additionally, controls involving non-EV fractions can aid in distinguishing specific EV effects. The impact of loosely tethered coronal elements on EV function should be also considered. Lastly, it's crucial to investigate the influence of factors like EV separation, storage, and formulation on EV activity. Potency assays can help identify the most relevant controls for pre-clinical and clinical studies [33, 65].

#### EV Dose Unit

The number of particles and the total amount of protein are widely used as the unit of EV dose, and these units may also be used in nonclinical studies. Meanwhile, these units are likely to be impacted by the purity and quality of EVs, leading to difficulty in obtaining stable test results in some cases. In such cases, if the active components have been identified, the content can be used as the unit of EV activity (e.g., equivalent to IL-2 XX mg and equivalent to miRNA XX copies), provided that EV preparations with similar identity were produced using the same process and cell source. It should be noted that even if two EV preparations have similar activity dose units, their therapeutic effectiveness may vary if they were produced using different processes. On the other hand, if the active component is unknown, the biological potency defined in the representative pharmacological study may be used as the unit.

#### Selection of Animal Species

Because most of the active components of EVs are proteins and nucleic acids and the effects of differences in animal species are significant, it is desirable to select animal species that exhibit the intended pharmacological action in humans when treated with the EV preparation. For biotechnological products, if the relevant animal species are not available, nonclinical evaluation can be performed using equivalent animal products, but for EV preparations, it is considered difficult to demonstrate the equivalence between clinical candidates and animal products. In such cases, it is essential to provide thorough scientific evidence to support the efficacy of the EV preparation. This evidence should be sourced from various published studies and data, illustrating the specific mechanisms of action and physiological effects that support the therapeutic potential of the EVs.

### Nonclinical Safety Studies

#### General Principles

Nonclinical safety studies of an EV preparation should be planned on a case-by-case basis after fully understanding its characteristics and conducted in accordance with Good Laboratory Practice (GLP) in principle. Because scientific progress and accumulation of experience in the field of EVs are constantly advancing, if new strategies and testing methods to replace the nonclinical safety evaluation described below are developed, they can also be used in nonclinical safety evaluation.

#### Evaluation Strategy

To conduct nonclinical safety assessment of EV preparations, it is imperative to evaluate the safety profile upon human administration, considering both on-target toxicity, which results from the intended pharmacological action and off-target toxicity, which is not directly related to the intended pharmacological action, as is done with other drugs.

- On-target toxicity

The on-target toxicity of EV preparations is considered to be induced by proteins, oligonucleotides (mRNA, miRNA, and DNA), etc. contained as active components, and it should be evaluated based on the mode of action. Therefore, for evaluation of on-target toxicity attributable to proteins, mRNA and DNA, and miRNA, " Preclinical safety evaluation of biotechnology-derived pharmaceuticals" (ICH S6) [66], "Guideline on Quality, preclinical and clinical aspects of gene therapy medicinal products" [67] and "Guideline for preclinical safety assessment of oligonucleotide therapeutics" [68] can be used as references, respectively.

b) Off-target toxicity

It is considered that native EV preparations consist of various types of EVs, and proteins and nucleic acids in each EV have high target specificity. Thus, the EV preparations are considered unlikely to cause off-target toxicity compared with chemical entities. For EV preparations from cells with minimal concern about safety in humans, that have already been utilized in cell therapy products, off-target toxicity concerns are considered to be low. Therefore, in the case of native EV preparations, it is not required to conduct an independent study for off-target toxicity, as it is sufficient to evaluate it in nonclinical safety studies for on-target toxicity.

On the other hand, off-target toxicity of engineered EV preparations with chemical modifications (e.g., modification of nucleic acids and conjugation) could be a concern when administered to humans, and sponsors should evaluate it on a case-by-case basis using scientifically appropriate approaches. If any chemical entities whose safety in humans has not been assured (e.g., chemical modification by bioconjugation) are to be used in a engineered EV preparation, nonclinical safety studies should be conducted with reference to the "Guidance on non-clinical safety studies for the conduct of human clinical trials pharmaceuticals" (ICH M3) [69] or other appropriate safety documents related to chemical entities.

c) Carcinogenicity of EVs

It has been reported that EVs derived from immortalized cells contain numerous RNA and DNA sequences of retrotransposon, which can transfer the malignant phenotype of immortalized cells to normal cells via retrotransposons [70]. Furthermore, retrotransposons have been detected in FBS, which is a commonly used cell culture supplement [71–73]. Although it is difficult to evaluate the risks associated with retrotransposons in nonclinical studies, it is advisable to prioritize the use of cells with minimal safety concerns, such as those intended for cell therapy products in the manufacturing of EV preparations, and FBS should be avoided as much as possible. However, if it is indispensable to use cells with an unknown, safety profile, at least, retrotransposon activity in the cells should be measured to ensure low risk. In addition, the regarding carcinogenicity of EV preparations, including both native and engineered EV preparations using chemical entities without confirmed safety in humans, carcinogenicity studies of the chemical entities need not be conducted unless the preparations are continuously used for a prolonged period (6 months or longer) in the clinical use ("Guideline on the need for carcinogenicity studies of pharmaceuticals" (ICH S1A) [74]).

d) Impurities

It is important to reduce manufacturing process-derived impurities such as reagents used in the manufacturing of EV preparations as much as possible by using the information on manufacturing. It is considered appropriate to grasp the amounts of impurities remaining in the preparation as much as possible and evaluate the safety based on the nonclinical safety study results of the preparation, published data (e.g., toxicity profile, information on human endogenous substances, and experience of administration to humans), guidelines on impurities [75–79], and toxicological concepts (e.g., threshold of toxicological concern).

#### Design of Nonclinical Safety Studies

- Selection of animal species

In assessing the on-target toxicity of EV preparations, it is generally appropriate to conduct nonclinical safety studies in one animal species that exhibits the intended pharmacological action in humans when treated with the EV preparation. In the case of biopharmaceuticals and cell therapy products, when no animal exhibits the intended pharmacological action in humans, alternative models using analogous animal products are considered. However, evaluating the similarities between an EV preparation with an animal equivalent is considered complicated. Thus, when no animal exhibits the pharmacological action of an EV preparation, hazards caused by the on-target toxicity should be evaluated based on scientific evidence obtained from various published information for risk management in humans. In terms of *in vitro* alternative methods, including human organoids, the ICH M3 states that when these methods are validated and accepted by regulatory authorities, they can be used to replace current standard test methods. Therefore, if these methods are to be employed, early consultation with the regulatory authorities is recommended.

For native EV preparations derived from normal human cells, the concern for off-target toxicity when administered to humans is considered low, and it is supposed sufficient to evaluate in toxicity studies (e.g., single-dose toxicity studies and repeated-dose toxicity studies) in one animal species including rodents. On the other hand, when a chemical entity whose human safety has not been assured is used in an engineered EV preparation, two animal species (rodent and non-rodent) are generally required to evaluate off-target toxicity focusing on the chemical entities with reference to ICH M3.

b) Route, frequency and dose of administration

In nonclinical safety studies of EV preparations, in principle, it is desirable to administer EVs by the intended therapeutic route at a frequency equal to or more than that in clinical use. If the therapeutic route is not feasible, the influence of the difference in the administration route on the safety evaluation should be explained. Additionally, the aim of repeated dose studies is to identify toxicological changes resulting from the repeated administration of a test substance administered to both animals and humans. When an EV preparation does not accumulate *in vivo* and is unlikely to exacerbate toxicity findings after repeated administration to humans and animals, the necessity for repeated dosing is considered to be low.

Given that exosomes contain a wide variety of proteins and oligonucleotides and the biological activity of EV preparations is assumed to differ between animal species, addressing the quantitative human risk caused by the on-target toxicity of EV preparations is difficult, and it is necessary to identify the hazards. The dose levels of native EV preparations are considered evaluable in at least two groups, the control and treatment groups. On the other hand, if a chemical substance whose safety in humans is not identified is used in an engineered EV preparation, it is desirable to set multiple dose groups as for chemically synthesized drugs so that the dose–response relationship can be evaluated. Moreover, for all EV preparations, it is considered that the maximum dose should be as high as possible while considering the maximum tolerated dose (MTD), maximum feasible dose (MFD), and animal welfare (3Rs).

e) Reversibility

If serious toxicological findings are observed in nonclinical safety studies and extrapolation in humans is concerned, the reversibility should be evaluated with reference to ICH M3.

f) Toxicokinetics

For usual drugs, toxicokinetic data (systemic exposure data in toxicity studies) are collected to assess the extrapolation of nonclinical safety study results to humans. However, EV preparations are not homogeneous, and there is no established evaluation method for analyzing specific EVs separately. Thus, it is difficult to evaluate the toxicokinetics of EV preparations at present. In addition, given that biological activity differs between animal species for many EV preparations, it is considered unnecessary to collect toxicokinetic data in nonclinical safety studies to evaluate the human safety. On the other hand, if any chemical entity whose human safety has not been identified is used in an engineered EV preparation and there is a particular concern about its safety, the necessity of collecting toxicokinetic data focusing on the relevant chemical substance should be considered.

## Clinical Development

### PK/PD and Efficacy Assessment

PK/PD studies are important for the evaluation of maximization of therapeutic effects and minimization of adverse effects of drugs, but in light of the current situation where it is difficult to quantify the concentration of EV preparations in the body, it is not necessary to strictly determine the ADME/PK/PD, and it is realistic to analyze the relationship between the dosage and administration and effects/actions. Evaluation in clinical studies is desirable, but nonclinical studies or *in vitro* studies shall be conducted for items with difficulty. However, it should be noted that animal study data are not necessarily applicable to humans. The effects and actions to be evaluated are different depending on the disease or purpose as shown in the examples below. If there is a possibility that concomitant use of drugs with the same type of action or related action may enhance the main action, it is necessary to examine the interaction with the concomitant drug. In efficacy and safety evaluation in clinical development, immunogenicity and on-target/off-target toxicity should be considered as special properties of EVs and the development methods of low molecular weight compounds and RNA/protein drugs should be referred to. Regarding immunogenicity, if an EV preparation is administered repeatedly, attention should be paid because the effects/actions may be attenuated due to antibody development against molecules on EVs. Furthermore, attention should be paid to whether the intended dose is administered accurately because the concentration of EVs measured can vary based on factors such as the specific method used for quantification, the characteristics of the sample, and the environment in which the measurement is performed. Therefore, it's essential to carefully consider and control these variables to obtain accurate and reproducible results.

### Undesirable Immune Responses such as Allergy and Rejection

When EVs derived from allogeneic humans, non-human animals, or plants are administered to humans, risks due to undesirable immune responses such as allergy and rejection are assumed, but the significance of evaluating these risks in nonclinical studies in animals is limited. Thus, it is necessary to advance clinical studies carefully after considering measures to reduce risks related to undesirable immune responses in humans from the following viewpoints:

#### Immune Responses to EVs

The immunogenicity of an EV preparations differs depending on the origin of manufacturing cells. If it is derived from autologous cells, there is no need to consider the immunogenicity of the EVs themselves. In the case of allogeneic human origin, immunogenicity resulting from the incompatibility of the histocompatibility complex expressed by EVs themselves, especially MHC class I and II of surface antigens, should be considered. As long as they have the potential to transfer or maintain the immune system in its regulatory state, they should be tolerated. Focusing on pregnancy, embryos are well tolerated as long as the maternal immune system remains in this regulatory state. However, when administering EVs of allogeneic human origin to the maternal body during pregnancy, there is a potential for the induction of pro-inflammatory processes. If such processes occur, it is readily conceivable that they could result in severe complications for the fetus, since EVs mediate fetal-maternal communications during pregnancy [80]. Caution is necessary when using EVs for disease treatment during pregnancy, depending on the nature of the originating-cells in the allogeneic setting. The safety of administration during pregnancy has not been established. Therefore, it should only be administered if the therapeutic benefits are deemed to outweigh the risks.

No immunosuppressants are used in treatment with cell therapy products or cell processing products that are expected to have a short-term effect, such as MSC, a therapeutic drug for acute graft-versus-host disease [81]. In addition, no description requiring actions relating MHC (matching of MHC) is found at present in the investigation of EV-related clinical studies using public information such as the US clinical trial registration database (ClinicalTrials.gov). Furthermore, considering that transfusion of platelets expressing human leukocyte antigen (HLA)-A and HLA-B (so called random platelet transfusion) has been performed globally for a long time, serious immune responses via MHC are unlikely to occur in clinical application of EV preparations and safety concerns are considered to be low.

On the other hand, in treatment with allogeneic human cell-derived cell therapy products and cell processing products for which long-term engraftment is expected, immunosuppressants are used, and long-term engraftment requires control of rejection due to MHC mismatch [82]. Given that EVs are rapidly metabolized, immunosuppression may be necessary in instances of repeated administration of allogeneic EVs. To reduce immune responses such as acute and chronic rejection, the same approach in hematopoietic cell transplantation, i.e., matching of MHC class I A, B, and C loci and class II DR locus in the direction from the recipient (patient) to the donor (EVs administered) (so-called the host versus graft [HVG] direction) might be useful. It is important to consider the necessity of matching MHC class according to the purpose for which EV preparations are applied.

#### Immune Responses to Impurities other than EVs (e.g., Antibiotics and Medium Components)

For impurities (e.g., antibiotics and medium components) contained in therapeutic preparations using EVs, risks such as allergy are considered, and treatment with an appropriate combination of drugs such as antihistamine, anti-inflammatory drug, and antipyretic analgesic before the use of EV preparations is also an option to avoid the relevant risks. At least, when the relevant preparation is administered to humans, appropriate clinical safety management will be necessary, for example, fully explaining and calling attention to subjects and investigators after reducing the relevant risks as much as possible (see Section "Impurities in EV Preparations"). Impurities in EV preparations include unintended EVs and EV variants, viruses/virus-like particles, microorganisms, and mycoplasma, medium components/reagents, airborne particles and artificial microparticles. For reducing the risks of immune responses to such impurities, the quality issues are needed to be considered. It is important to minimize their inclusion/contamination by design and control of appropriate manufacturing processes. In addition, it is desirable to avoid the use of animal-derived raw materials (including humans) to the extent possible and use of reagents and recombinant proteins free from animal-derived components is recommended.

#### EV-Induced Immune Responses

The intended pharmacological action (on-target effect) may be caused by immune responses induced by EVs unlike the cases stated in Section "Immune Responses to EVs" for responses caused by the immunogenicity of EVs. However, it should be noted that the response may exhibit excessively (on-target toxicity). For example, there is a possibility for the onset of infection caused by excessive immunosuppression because the intended pharmacological effect of MSC-derived EVs is immunosuppression. Conversely, the intended pharmacological effect of DC-derived EVs is to activate the immune response, and thus prophylactic and therapeutic use of immunosuppressants, etc. for excessive immune response (e.g., cytokine storm) should be considered.

### First-in-Human Study Design

Ensuring safety in humans is an extremely important issue in clinical studies. In particular, in a clinical study in which a drug is administered to humans for the first time (FIH study), it is necessary to carefully determine the FIH study design including the initial dose, dosing interval, and risk management method based on all available information.

Because many EV preparations are expected to induce biological reactions via highly species-specific molecules such as proteins and nucleic acids (mRNA, miRNA, and DNA), there is a limitation in estimating the initial dose in the FIH study based on the results of nonclinical studies in animals. Thus, if the results of clinical studies of similar EV preparations have already been obtained, it is recommended to draw up an FIH study design including the initial dose by utilizing the information as much as possible. On the other hand, if no information on similar EV preparations is available, the initial dose in the FIH study should be determined from the perspective of both on-target and off-target toxicity using the nonclinical data of the EV preparation. From the standpoint of on-target toxicity of EV preparations, if a critical safety concern due to excessive pharmacological activity is expected when administered to humans, it is necessary to determine the initial dose by estimating the minimal anticipated biological effect level (MABEL) in clinical studies of the EV preparation with reference to the "Revised guideline on first-in-human clinical trials" [83]. Meanwhile, if there is no critical concern about on-target toxicity of the EV preparation, it is not necessarily required to calculate the MABEL. In addition, if a safety concern is expected from the viewpoint of off-target toxicity, the initial dose should be determined based on the no observed adverse effect level (NOAEL) calculated based on the general toxicity studies of the EV preparation. Based on the above, in the FIH study, it is appropriate to determine the initial dose by selecting a dose determined based on the MABEL and/or NOAEL, whichever is lower and by multiplying the dose by an appropriate safety factor. For other points to consider in conducting the FIH study, the FIH study guidance and the "Questions and Answers (Q & A) on Guidance for Establishing Safety in First-in-Human Studies during Drug Development" [84] would be useful as a reference.

## Conclusions

This review summarized recent discussions among EV research experts in Japan, focusing on the advancement of EV-based therapeutics. The primary objective of this article is to stimulate and support the implementation of clinical research using EVs within Japan. However, it is our hope that this article will facilitate the development of novel EV-based therapies by providing guidance to researchers, companies and regulatory reviewers not only in Japan but also globally. However, it is important to note that the field of EV technology is rapidly evolving, with a multitude of emerging advancements. Consequently, this document should be periodically reviewed and updated to incorporate the latest technological developments.
